# Early Rupture of Membranes

**Published:** 1878-04-20

**Authors:** W. S. Posey

**Affiliations:** Sulphur Bluff, Texas


### SOUTHERN MEDICAL RECORD.

VOL. VIII. ATLANTA, GA., APRIL 20, 1878. NUMBER 4.

EDITORS:

THOMAS S. POWELL, M. D. W. T. GOLDSMITH, M. D. R. C. WORD, M. D.

*R. C. WORD, M. D., Managing Editor.*

SUBSCRIPTION—$2.00 PER ANNUM, IN ADVANCE.

☞ All Communications and Letters on Business connected with THE RECORD, for 1877 and 1878, must be addressed to the Managing Editor.

### *ORIGINAL AND SELECTED ARTICLES.*

### EARLY RUPTURE OF MEMBRANES.

By W. S. POSEY, M.D., of Sulphur Bluff, Texas.

I am only a plain country practitioner, and it is not without fear of being considered impertinent that I here urge an objection to a time-honored practice of the fathers of medicine and their illustrious successors.

On page 27 of the January number of the RECORD, we find a paragraph on early rupture of membranes, closing with these words: "Meddlesome midwifery is bad." I have heard this quotation over twenty-five years without knowing the author or understanding the range or scope of its meaning. It was a favorite expression with my highly gifted preceptor, late Professor John M. Watson, of the University of Nashville. "Meddlesome midwifery!" May I not say with the same propriety, meddlesome surgery, or meddlesome practice in the common diseases of our country? The surgeon skillfully plunges the knife to the hilt for the relief of severe injuries or malignant diseases, without the fear of any one saying "meddlesome." With like promptness and skill should the obstetrician try to "improve on nature's processes" when nature is inadequate to the task and his patient under volcanic throes of labor. Why has obstetrics been reduced to a science and taught as an art, if we are not to "seek to improve on nature's tardy and unsatisfactory processes?" It should be regarded a duty as well as a privilege, by all scientific and honorable means and plans, to alleviate the parturient woman. With this object in view, I frequently rupture the membranes early, and I think I have saved quite a number from instrumental labor by so doing. I have noticed for years a circumstance connected with the amniotic membranes that I have not seen on record, viz: in their circumferences I have found the membranes so small that though the fœtus and liquor amnii would distend them to their utmost capacity, they never bag nor portrude through the os, and when the pains come on, though they feel as tight as a drumhead, they fail to press upon or distend the os. The result of this condition is that the uterine muscular fibres are powerless, not by reason of over-distention or dropsy of the amnion, but rather want of size and elasticity of the uterine membranes. This is a different case to that of dropsy of the amnion. An excessive quantity of liquor amnii may likewise cause lingering labor, the uterine fibres being distended beyond the point of contraction. To distinguish the condition to which I allude, introduce the finger or fingers. The vagina and perineum are in a state of distensibility. The sacro-ischiatic and coccygeal ligatures flacid and soft, the os is dilated to the size of a silver dollar or more. Turn the finger around and upwards, and you feel a tense slick elastic membrane, firm and tight upon pressure. When a pain comes on, the child's head descends, the waters splash and separate and pass up, while the head presses feebly on the os. The pains simulate cramp. The old ladies will tell you the pains are "working up, doctor, they must work down." Hours are whiled away and no progress is made in the labor, but dreadful suffering to the exhaustion of the vital forces. Here is nature's process forcibly exemplified.

*Treatment.*—Lay aside your *prejudice*, ergot and teas, rupture the membranes and let the woman get down to her work. The waters will flow, the uterus will contract, the fibres will get up to their responsible duty, the head will engage the straits, and soon delivery will be accomplisned.

We offer an imperfect report of two cases, from quite a number, as an illustration of the propriety of early rupture of the membranes:

CASE NO. 1.

Mrs. ——, æt. 22, in her second confinement; utero gestation completed; had hooping cough; took a chill at 9 o'clock a. m. January 20th; saw her at 5 o'clock p. m. same day; fever very high; skin hot and dry; tongue coated; dreadful pain in her head; light eclampsia; pain in back, which extends around the abdomen; pulse quick and small; vagina hot and dry; os not dilated. Four o'clock, January 21, her fever had declined a little; headache a little better. True labor set in from this hour until 9 o'clock, and progressed very slowly and with much suffering. To fight against so many enemies—hooping cough, fever, eclampsia, pressed us to our wits' end. Nine o'clock, labor pains bearing down, vagina relaxed and well lubricated, os dilated to size of silver dollar, thin and yielding, and the membranes protruded about one inch and a half. I ruptured the membranes and lifted the head of the child with my finger, and the waters flowed freely, and the woman was safely delivered in twenty-seven minutes after the membranes were ruptured.

CASE NO. 2.

Called to see Mrs. —— February 14, 23 years old, second confinement; utero gestation completed; had diarrhœa for two weeks, which resulted in dysentery; pulse 110; tongue coated; high fever; skin hot and dry; pains all over, but worse in head and back; vagina and perineum relaxed, hot and dry; os dilated so I could press my finger through; uterine pains tolerably strong. My treatment suspended the labor until the next day, 15th, when it was apparent that I could no longer await the operations of nature, the patient being much exhausted and her general constitution no better. The expulsive pains were very severe, but the membranes did not protrude or bag; they were tight as above described. I introduced my fingers inside the os uteri, and ruptured the membranes. A very large quantity of liquor amnii escaped, to the indescribable relief of my patient, and the labor ended in fifty-two minutes, placenta and all.

The reader will see that I have attempted to state facts, without arguing questions, in the fewest words possible, on account of the limited size and valuable space in the RECORD. What a pity that this most excellent and popular of all the valuable medical periodicals in the South could not be enlarged and still more widely sustained.

